# A large-scale expert-annotated 3D image dataset of dermal tissue macrophages

**DOI:** 10.1038/s41597-026-08556-7

**Published:** 2026-10-20

**Authors:** Amirhossein Kardoost, Nisreen Ghanem, Tianru Li, Julia Kolter, Carsten Marr

**Affiliations:** 1 Institute of AI for Health & Helmholtz AI, Computational Health Center, Helmholtz Munich – German Research Center for Environmental Health, Neuherberg, Germany; 2 Munich Center for Machine Learning (MCML), Munich, Germany; 3 Institute for Infection Prevention and Control, Medical Center and Faculty of Medicine, University of Freiburg, Freiburg, Germany; 4 Faculty of Biology, University of Freiburg, Freiburg, Germany; 5 Department of Mathematics, Technical University of Munich, Munich, Germany; 6 Department of Medicine III, Ludwig-Maximilian-University Hospital, Munich, Germany; 7 Department of Physics, Ludwig-Maximilian-University, Munich, Germany; 8 German Cancer Consortium, DKTK, partner site Munich, Germany

## Abstract

Tissue macrophages are highly versatile immune cells whose morphologies shift dramatically depending on their activation state and tissue environment. This morphological plasticity reflects their diverse origins and functions but also poses significant challenges for both manual and automated image analysis. In the skin, macrophages display particularly intricate shapes, ranging from rounded to highly elongated, irregular, and non-convex forms. We developed a deep learning–based approach for the automated identification, 3D segmentation, and quantitative morphological profiling of dermal tissue macrophages in mice, facilitating large-scale analysis of cellular volume and elongation. This allowed us to build a dataset of 3D-segmented dermal tissue macrophages, comprising 947 single cells. To ensure accuracy, automatically generated segmentations were refined using a custom-designed Napari plugin. To illustrate technical validity and potential reuse, we quantified macrophage volume and elongation in adult steady-state and five-day post-*Staphylococcus aureus* infection samples, showing that the dataset captures biologically interpretable morphological variation.

## Background & Summary

Macrophages are complex tissue-resident immune cells that exhibit diverse morphologies, even among populations within the same tissue. Their functional repertoire extends far beyond pathogen clearance, encompassing vital roles in tissue morphogenesis, homeostasis, repair, and immunoregulation^1^. Their profound plasticity facilitates this functional versatility – the capacity to dynamically alter their phenotype in response to microenvironmental cues^2–4^. The skin, the body’s primary barrier, harbors a sophisticated immune network in which macrophages are indispensable regulators. The dermal compartment, in particular, contains a diverse and spatially organized macrophage population that is critical for both immune surveillance and structural maintenance^5–8^. In response to different environmental stimuli, dermal macrophages can polarize towards a wide spectrum of phenotypes and acquire critical functions^7,9,10^. To cope with this degree of plasticity, multiple classification systems have been proposed based on transcriptomic signatures^11^, surface marker expression, or functional attributes^12^. However, accurate morphological characterization of macrophages remains challenging due to the lack of comprehensive 3D datasets, the intrinsic difficulty of detecting individual cells in complex tissue environments, and the extensive morphological variability they possess^13–15^.

Macrophage segmentation has been previously performed in 2D using a filter-based method, which allows for fully automated macrophage counting in whole tissue sections^16^. This pipeline identified macrophages by applying median filtering, denoising, adaptive thresholding, and morphological feature filtering. It was evaluated on 50 regions of interest (ROIs) and 44 whole tissue sections, detecting macrophages at densities of 350–1700 cells/mm^2^ and showing strong correlation with manual counts. Machine learning-based approaches leverage cell morphology and multivariate feature profiles. In a previous study, 2D fluorescence microscopy images of stained *in vitro* cell cultures were segmented using CellProfiler, and quantitative morphometric features were measured^17^. Differently polarized macrophages were then identified based on cell morphology alone and could be distinguished from naive macrophages and monocytes. Another study identified and segmented overlapping macrophages in 541 time-lapse 2D fluorescent microscopy frames obtained from embryos of *Drosophila melanogaster*^18^. Here, individual cells were separated through junction-based segmentation and quantified with extracted shape features. Segmentation performance was evaluated on 10 manually annotated frames, focusing on accuracy rather than total cell number. Geng *et al*.^18^ combined 3D light-sheet microscopy with slice-wise Mask R-CNN segmentation^19^, followed by post-processing to reconstruct crown-like structures in 3D. This approach enabled volumetric analysis of macrophage-rich adipose microenvironments, but did not apply 3D instance segmentation of single cells. Thus, although macrophage segmentation has been studied in 2D time-lapse microscopy and macrophage-associated tissue structures have been quantified in 3D, curated workflows and datasets for single-cell 3D segmentation of tissue macrophages in volumetric tissue z-stacks are missing so far. To address this gap, we provide a curated dataset of 3D dermal macrophage segmentations from mouse skin tissue z-stacks. The dataset can support benchmarking of 3D instance segmentation methods, development of weakly supervised annotation workflows, quantitative morphology analysis of tissue-resident immune cells, and validation of image-analysis pipelines for complex whole-mount microscopy data.

## Methods

### Ethics statement

All animal experiments were approved by the Federal Ministry for Nature, Environment, and Consumer Protection of the State of Baden-Württemberg (registration number: G-20/083). Mice were bred and maintained at the CEMT animal facility of the University of Freiburg. Infected mice were anesthetized by isoflurane before the intradermal injection, and all mice were euthanized by carbon dioxide inhalation. Skin tissue was obtained from ears by excising them at the hairline for downstream analysis. All experiments were performed in accordance with the relevant national, federal, and institutional regulations. This study is reported in accordance with the ARRIVE guidelines for animal research^20^.

### Sample preparation & imaging

**Fig. 1:**
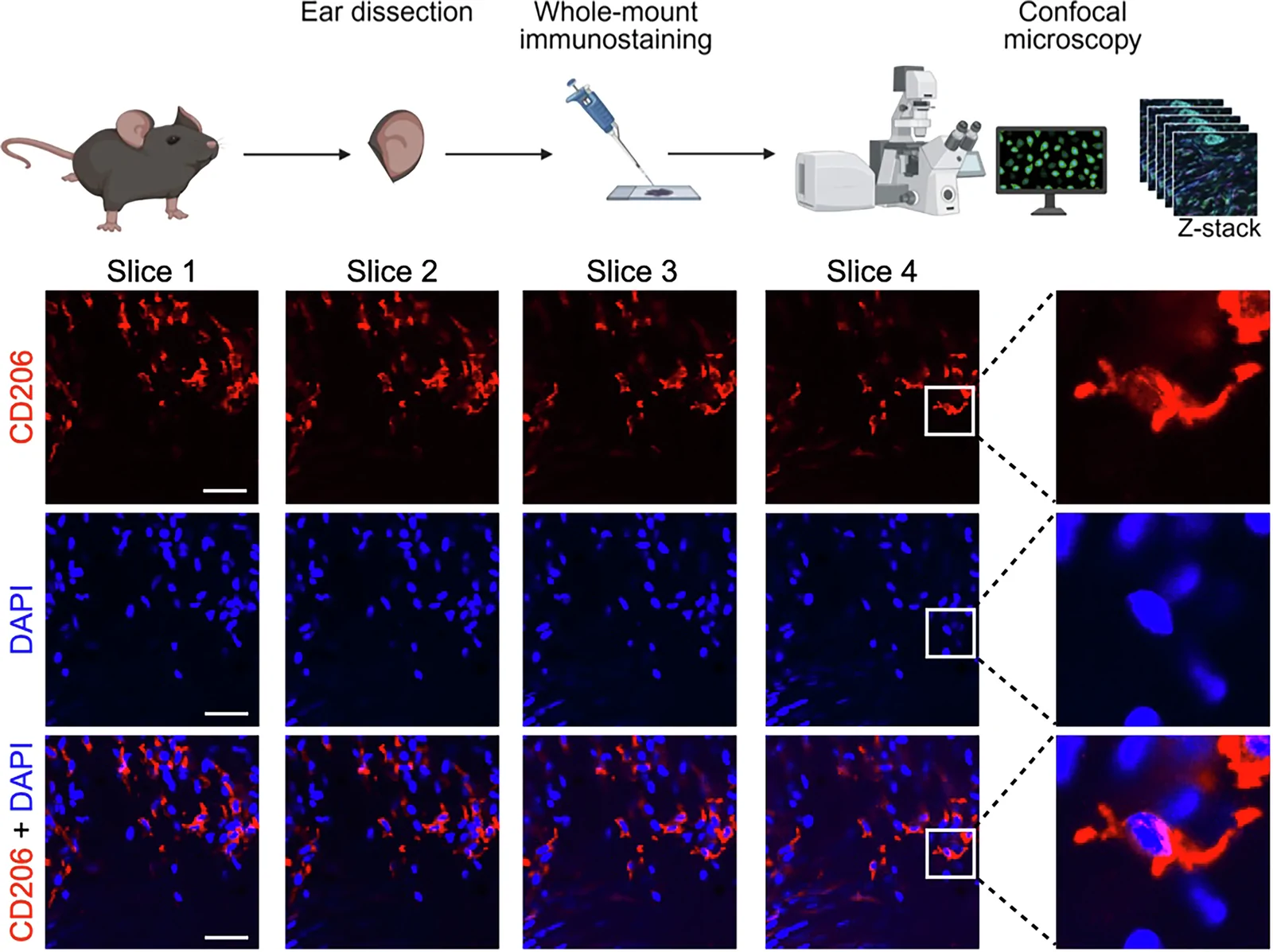
Adult ear skin whole-mount immunostaining and imaging. (Top) Workflow from ear dissection through confocal microscopy imaging and z-stack creation. (Bottom) Representative confocal images of the first four slices from a 22-slice tissue z-stack, illustrating macrophage-specific CD206 expression in red, DAPI nuclear staining in blue, and merged channel views. High-magnification insets from the same slices show the complex morphology of CD206-positive macrophages and the subcellular localization pattern of CD206. Scale bar represents 50 µm.

All mice were on a C57BL/6 genetic background, bred at the CEMT animal facility of the University of Freiburg. Mice were bred under specific pathogen-free conditions in the animal facilities of the University of Freiburg and housed in groups of up to five mice. Mice were kept in 12 h light/dark cycles, and food and water were provided ad libitum. Adult mice were between 6 and 8 weeks of age, and both male and female mice were used for experiments. For whole-mount preparations, mouse ears were separated into dorsal and ventral halves and incubated overnight at 4 °C in the dark in 1% paraformaldehyde in 24-well plates (Fig. 1). Samples were then incubated in wash buffer (1% bovine serum albumin, 0.1% Triton X-100, 4 °C). After mechanical removal of cartilage, the ears were stained with a wash buffer containing anti-CD206 AF488 (BioLegend), anti-F4/80 PE (BioLegend), Anti-Collagen-IV (Abcam), and DAPI overnight at 4 °C, followed by three washes. Dermal macrophages express the transmembrane receptor CD206, which was used to identify them in tissue samples in combination with F4/80^8^. Collagen IV was used to stain the basement membrane of structures like the blood vessels and the neurons for tissue context, and DAPI to detect cell nuclei (Fig. 1). Finally, tissues were mounted on microscopy slides using ProLong Diamond, dried overnight, and imaged with a Zeiss LSM 880 AiryScan confocal microscope with a 40 × objective. Z-stacks of 22 slices were acquired with 1024 × 1024 pixels per slice, where in-plane pixel size was ~0.21 µm and between slices was ~1.51 µm.

### Bacteria culture

*Staphylococcus aureus* Newman was grown in Luria-Bertani (LB) broth medium to the exponential growth phase, washed once with Dulbecco’s PBS (Phosphate Buffered Saline), and then resuspended in 10 ml PBS. Bacterial concentrations were determined using an optical spectrophotometer (600 nm, OD600).

### Mouse model of intradermal skin infection

Mice were anesthetized with isoflurane. A suspension containing 10^7^ colony-forming units (CFUs) of *Staphylococcus aureus* Newman in 10 µl of phosphate-buffered saline (PBS) was intradermally injected into the ear pinnae of anesthetized mice using a 30-gauge needle and a U-100 insulin syringe (BD Biosciences). Five days post-infection, mice were euthanized, and the ears were excised at the hairline for downstream analysis.

Whole-mount skin macrophages displayed a broad spectrum of morphologies in 3D, ranging from spherical to highly irregular, non-convex forms (Fig. 1).

### Macrophage detection

**Fig. 2:**
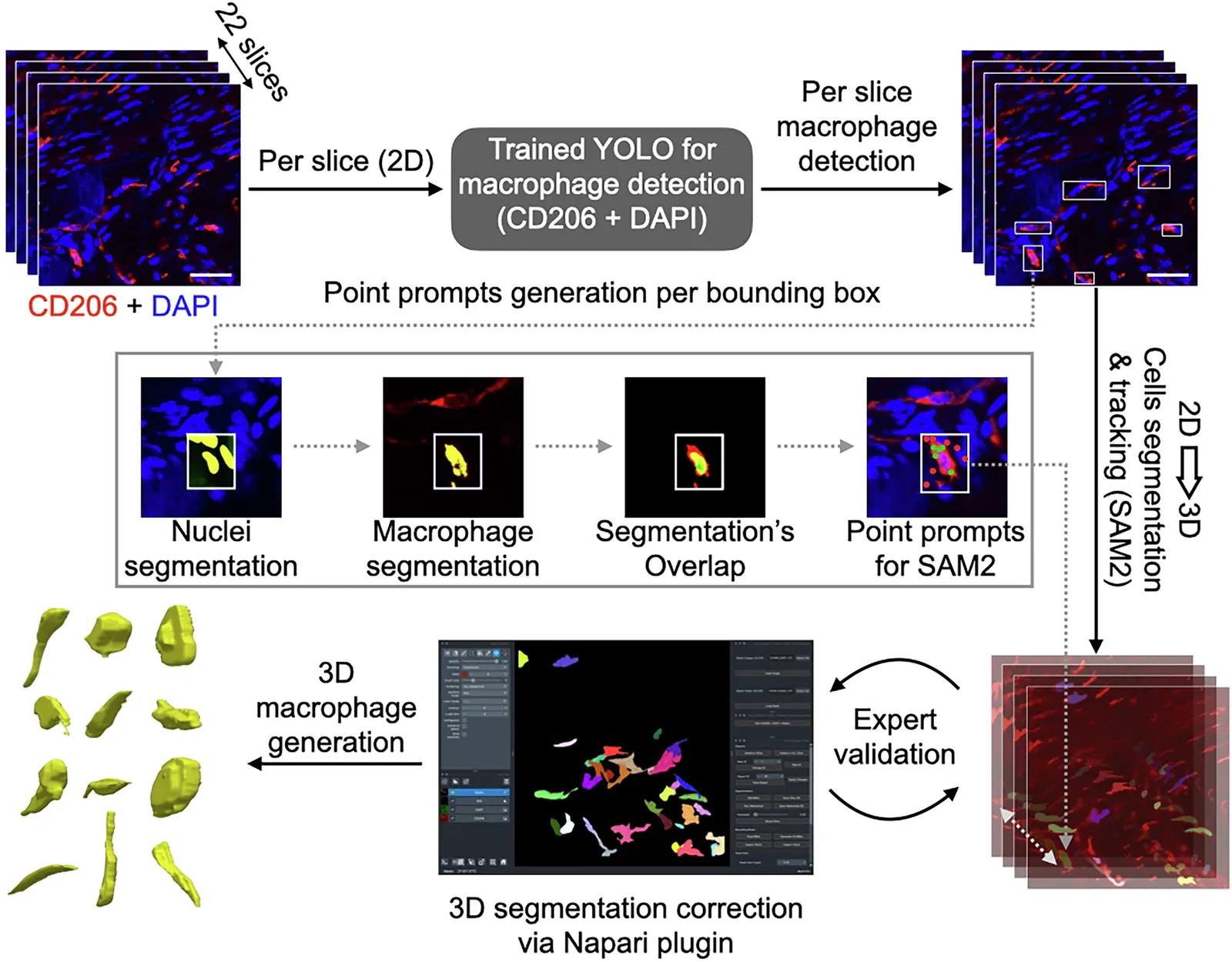
Expert-guided 3D macrophage segmentation and analysis workflow. Starting from raw z-stack volumes, single cells were detected as bounding boxes using YOLO^21^, followed by initial macrophage segmentation via Otsu^22^ thresholding. Nuclei were segmented with Cellpose^23^, and the overlap between macrophage and nuclear masks was used to generate positive (green) and negative (red) point prompts for SAM2^24^. These prompts guide slice-wise segmentation and tracking with SAM2 to support consistent macrophage identification across slices. All results were reviewed and corrected by experts in a Napari-based plugin, and the final 3D macrophage objects were reconstructed after validation. Scale bar represents 50 µm.

We developed a computer-assisted 3D macrophage segmentation and analysis workflow (Fig. 2) that integrates deep learning–based detection and segmentation with expert-guided validation and correction, enabling reliable identification and characterization of hundreds of single macrophages in 3D. To reduce the pixel-level annotation burden, we first performed 2D macrophage detection in the combination of DAPI and CD206 channels. The macrophage detection model was trained on 85 z-stack volumes, including additional internal imaging data used only for model development. These additional training volumes are not part of the public dataset release. The dataset described and released with this Data Descriptor consists of 31 expert-reviewed z-stack volumes and their corresponding 3D macrophage segmentation masks. For each volume, four slices (the 5th, 10th, 15th, and 20th) were selected to reduce manual bounding-box annotation costs while ensuring coverage of most macrophages within each volume. For each selected slice, macrophages (CD206) with clearly visible cell nuclei in the DAPI channel were annotated using a custom Napari plugin (Fig. 2). Manual bounding box annotation across all selected slices required approximately 8 hours in total per annotator. This resulted in 2,393 annotated bounding boxes. A nano YOLO (You Only Look Once)^21^ model, the smallest and fastest configuration with 129 layers and 3.2 million parameters, was trained on annotated bounding boxes.

### Segmentation & tracking

For each detected bounding box, an initial macrophage segmentation was generated on the CD206 channel using Otsu thresholding^22^, yielding a coarse segmentation mask. In parallel, nuclei within the same bounding box were segmented from the DAPI channel using the Cellpose^23^ model. Among the segmented nuclei, the nucleus with the maximum spatial overlap with the macrophage mask was selected (Fig. 2). Based on the combined macrophage and nuclear segmentations, the exact bounding box location was retained, and five random points were sampled from the union of the macrophage and nucleus masks as positive prompts, while eight random points were sampled outside the macrophage mask as negative prompts. These points, together with the bounding box, were provided as prompts to the SAM2^24^ model to propagate macrophage segmentation to the three preceding and three subsequent slices (Fig. 2). This procedure was applied to all detected bounding boxes, resulting in a preliminary 3D macrophage segmentation for each volume.

### Post-processing & quality control

The segmentation masks were independently reviewed by two annotators using a custom Napari plugin (Fig. 2). The plugin enables efficient inspection and refinement of 3D segmentations, including removal of spurious regions as well as correction or deletion of erroneous masks, thereby ensuring high-quality pixel-wise annotations. Cases involving macrophage occlusion or overlap across z-stacks were carefully examined and corrected through this interface, improving the consistency and accuracy of the final segmentation masks. The complete validation and refinement process again required approximately 8 hours per annotator.

### Morphological feature extraction

We considered volume and elongation as features for the 3D segmented macrophages. Volume reflects the total physical size of a cell, measured in cubic micrometers (µm^3^). Elongation was computed as the ratio between the longest and shortest principal axes of each segmented macrophage, estimated by principal component analysis of the physical voxel coordinates. This captured how much a cell deviated from a spherical shape in 3D, where a value of 1 corresponds to a perfect sphere and higher values indicate greater elongation.

## Data Records

The publicly released dataset consists of 31 fluorescence microscopy z-stack images and corresponding 3D instance segmentation masks of 947 individual macrophages stored in TIF format, and is available at https://doi.org/10.82296/hmgu-nefeli.dz19x-m8d59^25^. Additional private z-stack volumes were used during model development but are not included in the released dataset. The dataset is organized into two main directories, images and masks, each containing two subdirectories: AdultMice and AdultMice-Infected. The AdultMice directory contains 20 folders, each corresponding to one z-stack volume, e.g., Coll4AF633_F480PE_CD206AF488_DAPI_1. Folder names encode the imaged channels and their order, while the final integer denotes the image ID. Each folder contains the corresponding TIF z-stack volume or segmentation mask following the same naming convention. The AdultMice-Infected directory follows the same structure and contains 11 folders representing samples collected five days after Staphylococcus aureus infection. Each image TIF file contains a five-channel z-stack with the following channel order: Collagen IV, F4/80, CD206, DAPI, and brightfield. The corresponding mask TIF files contain a single-channel z-stack, in which each 3D macrophage instance is represented by a unique integer ID.

## Technical validation

### Detection performance

The performance of the trained macrophage detection model was evaluated using group-based 5-fold cross-validation to prevent data leakage between image stacks. Splits were performed at the stack level, resulting in per-fold allocations of 51 stacks for training, 17 for validation, and 17 for testing. Table 1 summarizes macrophage bounding-box detection performance on the adult steady-state test set, evaluated at an intersection-over-union (IoU) threshold of 0.5.

**Table 1:** Macrophage bounding-box detection performance on the adult steady-state test set.

Set	Precision	Recall	mAP50	mAP50-95
Test	0.78 ± 0.01	0.77 ± 0.01	0.83 ± 0.02	0.43 ± 0.01

Macrophage bounding-box detection performance reaches a precision and recall of 78% and 77% on the adult steady state test set. For each metric, the mean ± std from 5-fold cross-validation is reported.

### Morphological validation

We investigated differences in morphological features between two conditions: adult mice in steady-state and adult mice at five days post-infection with *Staphylococcus*.

**Fig. 3:**
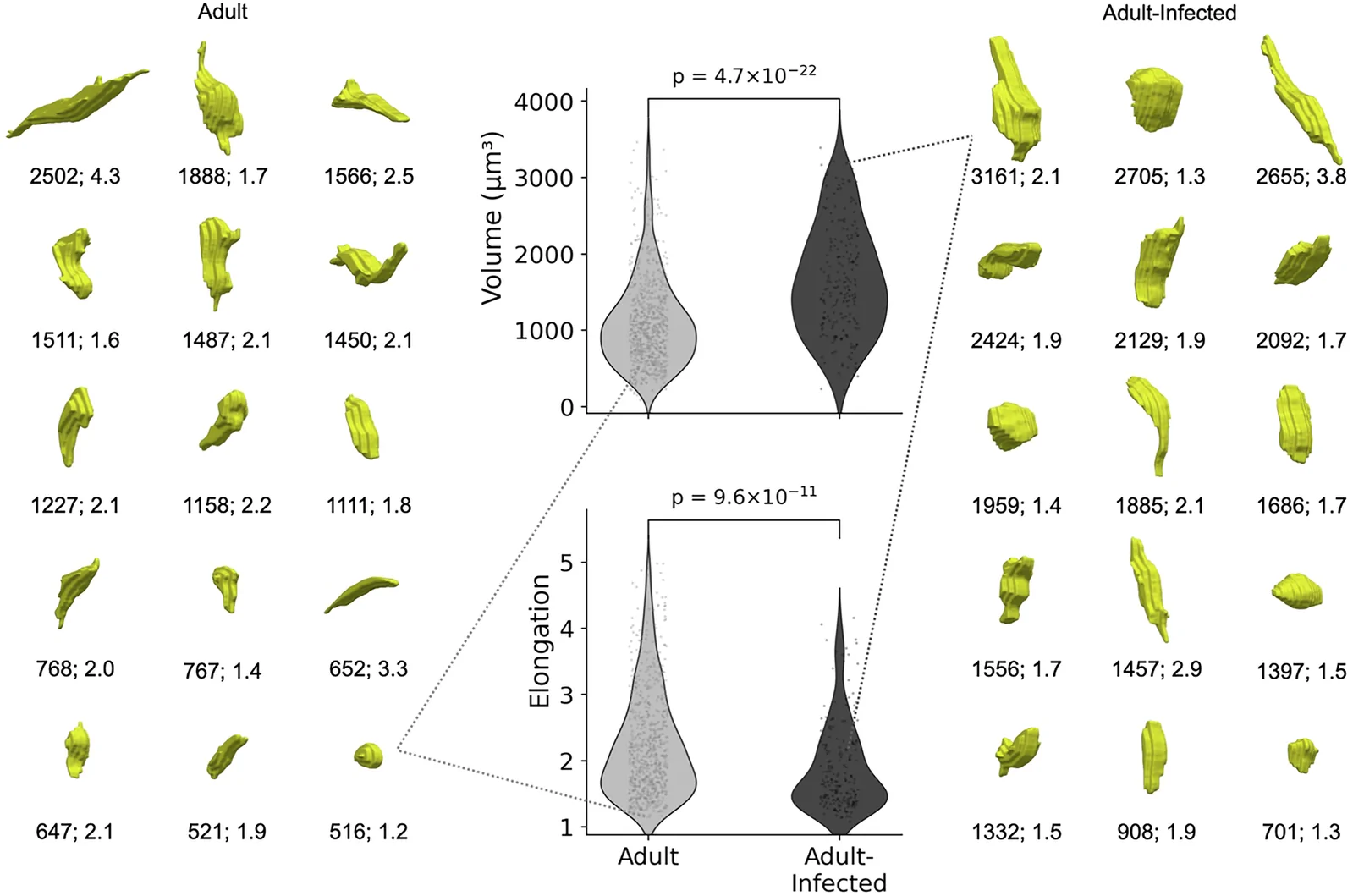
Macrophages from adult mice and adult-infected mice (five days post-infection with *Staphylococcus*) exhibit significant differences in 3D morphology. Individually segmented macrophages from adult-infected mice (237 single cells across 11 z-stack volumes) were significantly larger (1,604 ± 779 µm^3^ vs. 1,107 ± 595 µm^3^) and less elongated (1.93 ± 0.70 vs. 2.34 ± 0.99) compared with macrophages from adult non-infected mice (710 single cells across 20 z-stack volumes). Group comparisons were performed using a two-sided Mann–Whitney U test for both volume (p = 4.7 × 10^−22^) and elongation (p = 9.6 × 10^−11^). For each condition, a random subset of macrophages is shown and arranged from top-left to bottom-right according to decreasing cell volume. The values displayed below each rendering indicate the corresponding cell volume (µm^3^) and elongation.

As a case study, we compared macrophages from adult mice under steady-state conditions and adult-infected mice (five days post-infection with *Staphylococcus aureus*) in terms of volume and elongation. Three-dimensional morphological features were compared between 710 single cells from 15 adult mice across 20 z-stacks and 237 single cells from 4 infected mice across 11 z-stacks using a two-sided Mann–Whitney U test. Adult-Infected macrophages had significantly larger volume and lower elongation than adult macrophages in steady-state (both p < 0.001) (Fig. 3). The results are consistent with Rostam *et al*.’s^17^ work. Figure 3 also visualizes a random subset of the generated macrophages using 3D renderings, arranged in descending order of volume.

A limitation of our work is that the segmentation pipeline relies on two markers, DAPI and CD206, and that only cells detected in individual 2D slices are considered for subsequent 3D segmentation. Also, since segmentations are generated using SAM2, they currently need to be produced offline before being loaded into the Napari plugin.

## Data Availability

The dataset is available at https://doi.org/10.82296/hmgu-nefeli.dz19x-m8d59^25^. It contains 31 fluorescence microscopy z-stack images from adult steady-state and adult-infected mouse skin, together with corresponding expert-reviewed 3D macrophage instance segmentation masks in TIF format. The dataset is organized into images and masks directories, each containing AdultMice and AdultMice-Infected subdirectories. Image TIF files contain five-channel z-stacks ordered as Collagen IV, F4/80, CD206, DAPI, and brightfield, while mask TIF files contain single-channel z-stacks in which each macrophage instance is represented by a unique integer ID.

## Code availability

The code for the macrophage detection, segmentation, and 3D rendering pipeline is publicly available at: https://github.com/Amirhk-dev/macrophage-image-processor. The Napari plugin for macrophage visualization, post-processing, and manual correction is publicly available at: https://github.com/Amirhk-dev/macrophage-napari.
